# Supplementary material for: Orthobunyavirus spike architecture and recognition by neutralizing antibodies
Source: Nat Commun. 2019 Feb 20;10:879. doi: 10.1038/s41467-019-08832-8 (PMC6382863; doi:10.1038/s41467-019-08832-8)
Supplement: Supplementary file 1 — Supplementary Information [file 41467_2019_8832_MOESM1_ESM.pdf]

Supplementary Information

Orthobunyavirus Spike Architecture and Recognition by Neutralizing Antibodies

Hellert & Aebischer et al.

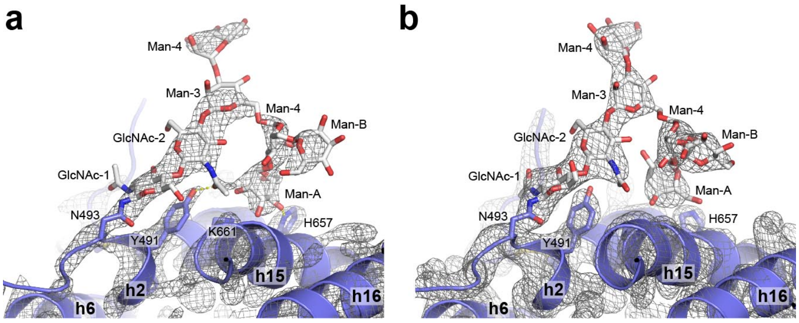

Supplementary Figure 1: The N493 glycan of SBV is partially ordered. Related to Fig. 1 and Fig. 5. **a** The N493 glycan in the crystal structure of the SBV Gc head domain in complex with scFv 1C11 and **b** in the crystal structure of the SBV Gc head domain in complex with scFv 4B6. In both structures the Y491 side chain contributes to the stabilization of the glycan. Residues H657 and K661 may also be involved, but owing to the rather low resolutions of 2.8 Å for 1C11 and 3.2 Å for 4B6, and owing to the anisotropy of both the datasets, their precise contributions remain unclear. The 2mFo-DFc electron density maps are contoured at 2.0 σ.

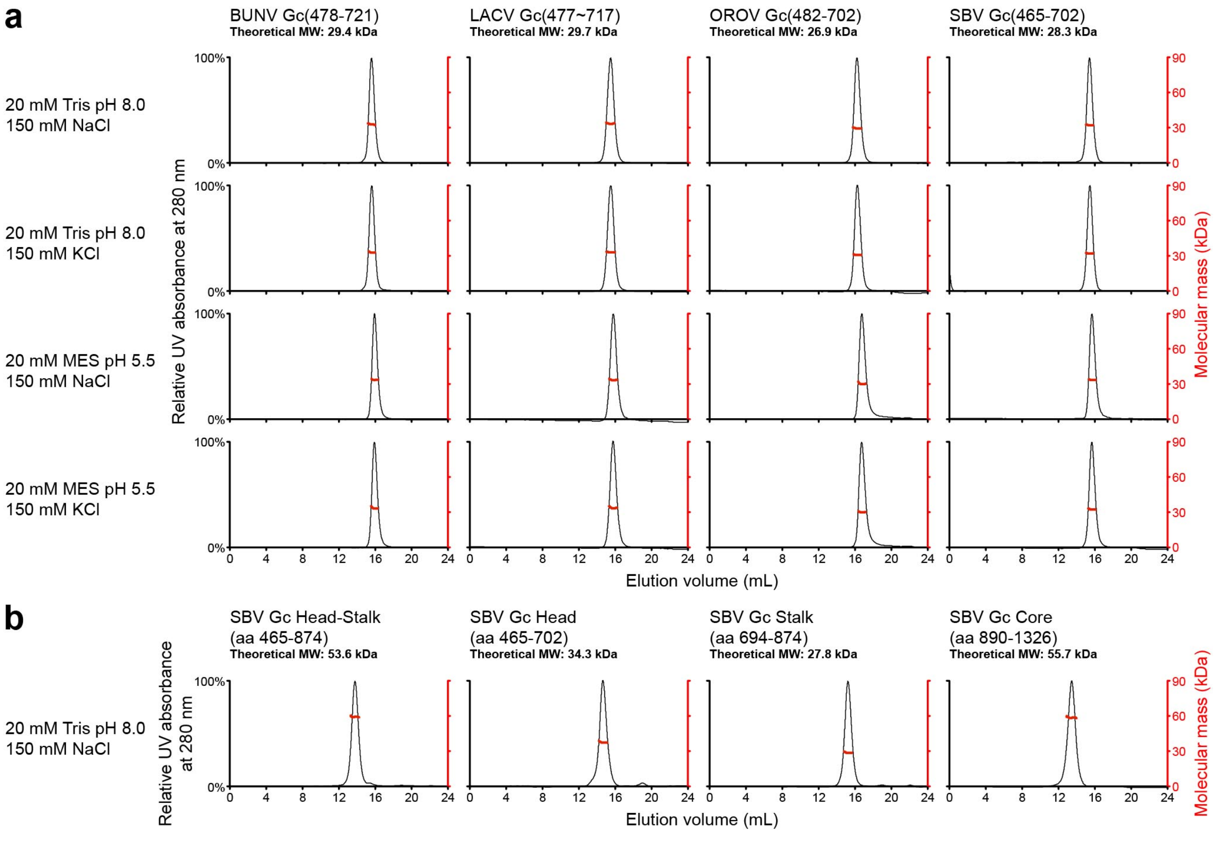

Supplementary Figure 2: Solution state of the recombinant protein fragments. Related to Fig. 2 and Fig. 3. **a** Size exclusion chromatography with multi-angle static light scattering analysis (SEC-MALS) for molecular weight determination of the recombinant Gc head domains of BUNV, LACV, OROV and SBV in the four buffers indicated on the left. The theoretical molecular weight (MW) of a monomer is given for each construct. The experimental MW measurement is shown in red for each peak and indicates monomers in solution for all constructs. **b** Equivalent analysis of the four constructs used for antibody depletion.

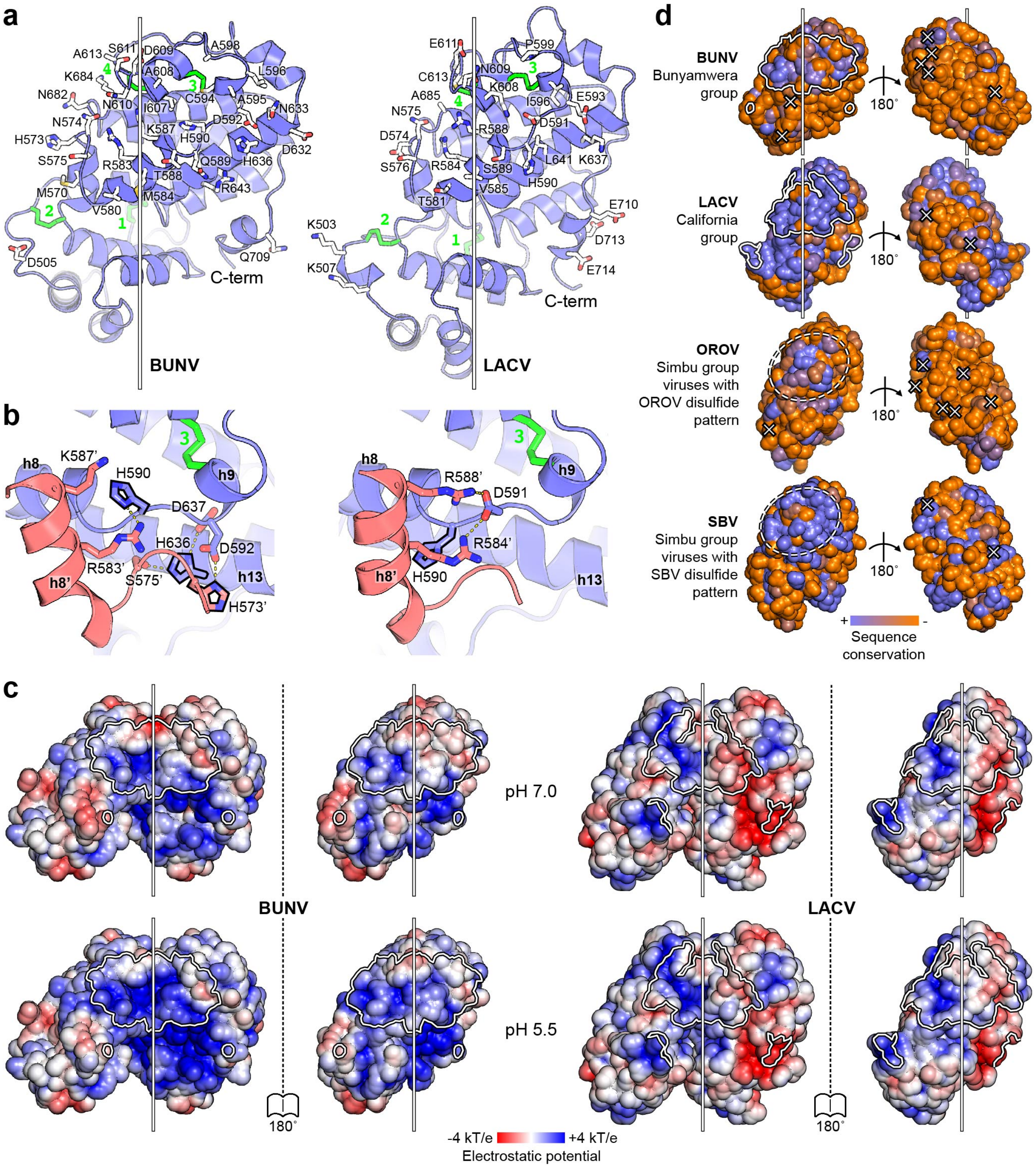

Supplementary Figure 3: Trimerization site of the Gc head domain. Related to Fig. 2. **a** Residues at the trimer interface of the Gc head domains of BUNV (left) and LACV (right). The threefold crystallographic axes are indicated as vertical bars. Side chains at the interface are shown with oxygen atoms in red and nitrogen atoms in blue. **b** Chemical environment of the histidine residues at the trimer interfaces of the Gc head domains of BUNV (left) and LACV (right). Two adjacent protomers are colored in red and blue. **c** Electrostatic potential on the trimer interface of the Gc head domains of BUNV (left) and LACV (right). The electrostatic surface potential was simulated for pH 7.0 (top) and pH 5.5 (bottom) using the PDB2PQR web server[1]. Negative charge is represented in red and positive charge is represented in blue. The trimer is shown in open book representation, with one protomer detached to the right to expose both sides of the trimer interface, which are outlined with a solid white line. The threefold

crystallographic axis is indicated as a vertical bar. **d** Relative surface conservation across closely related OBVs on the Gc head domain structures of BUNV, LACV, OROV and SBV (Supplementary Data 1). The relative surface conservation is color-coded with blue for high similarity and orange for high divergence. The experimental trimer interface areas for BUNV and LACV are outlined with solid white lines and the threefold crystallographic axes are indicated as vertical bars. The inferred trimer interface areas for OROV and SBV are outlined by dashed white lines. Predicted N-glycosylation sites among the compiled sequences are indicated as crosses.

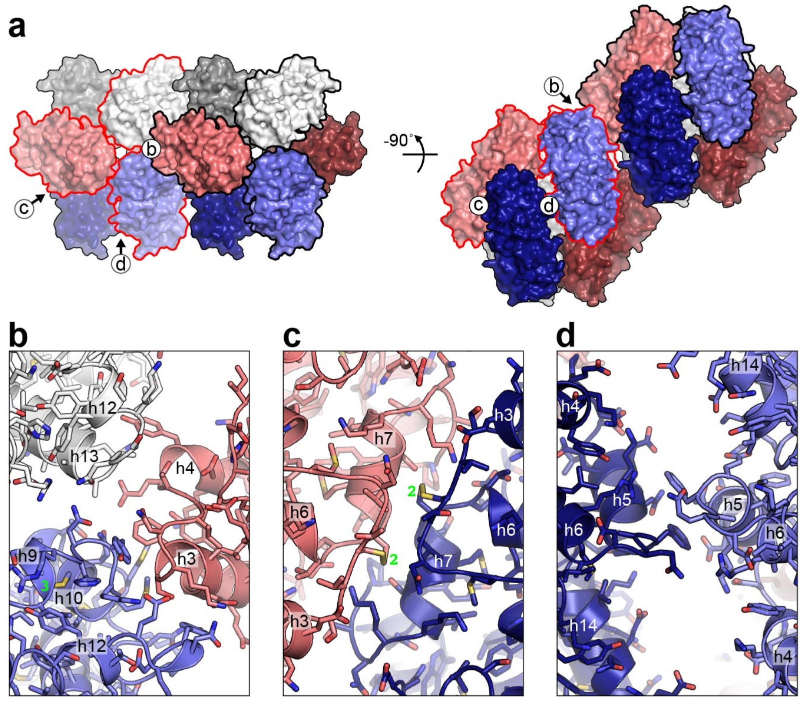

Supplementary Figure 4: Crystal packing of the OROV Gc head domain. Related to Fig. 2. **a** Top and side views on four open trimers as arranged in the crystal. The position of the three unique crystal contacts are denoted with lower case letters, and close-up views thereof are shown in the respective panels **b**, **c** and **d**.

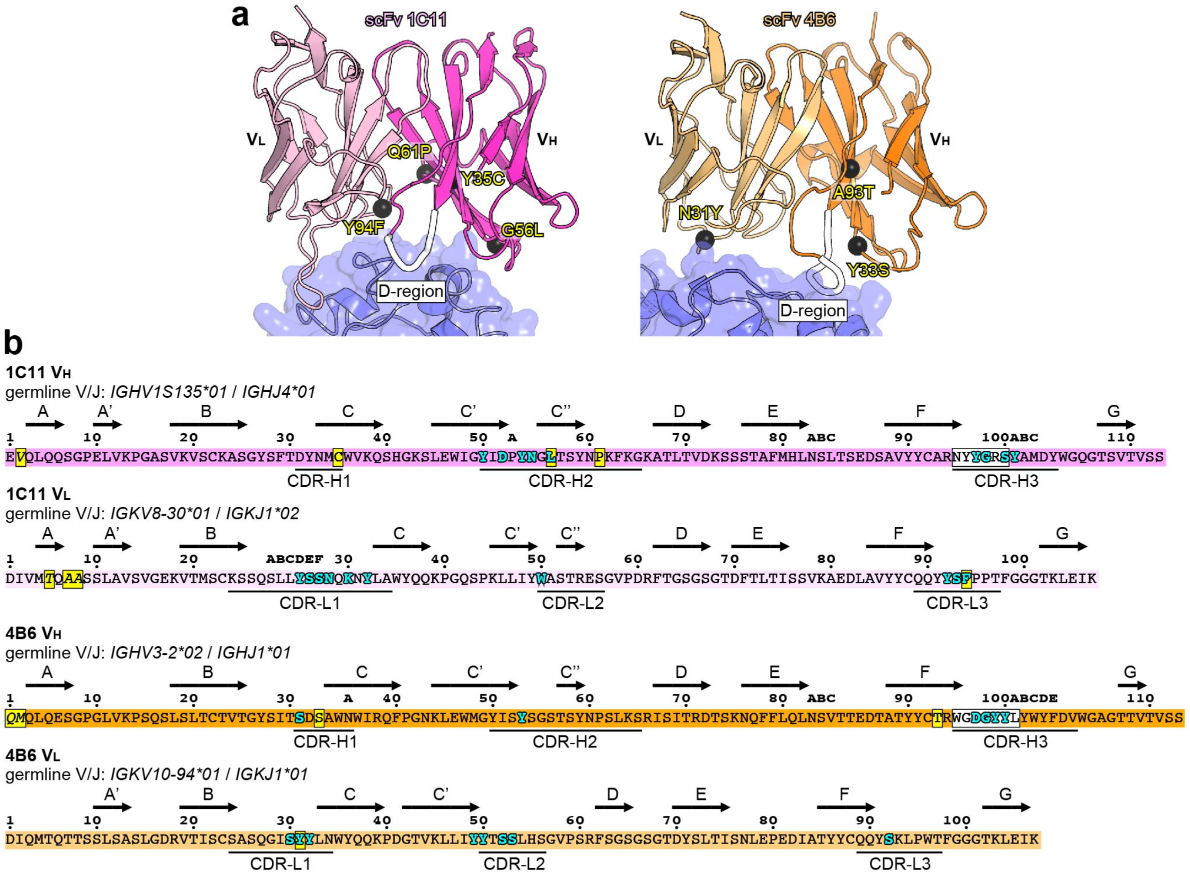

Supplementary Figure 5: Antibody sequences. Related to Fig. 5. **a** Crystal structures of scFvs 1C11 (right) and 4B6 (left) bound to the SBV Gc head domain (blue). The positions of point mutations in the antibodies with respect to the germline sequences are indicated as black spheres and labeled in yellow. The D-region of the heavy chains is shown in white. **b** Sequences of the V_H and V_L regions of 1C11 (top) and 4B6 (bottom). The V and J germline allele identifiers are provided above each sequence. β strands are indicated as arrows above the sequence. Point mutations with respect to the germline sequences are highlighted on yellow background. Mutations within the first eight residues of each sequence are likely introduced by mismatched sequencing primers and are indicated by italicized letters. Residues at the paratopes are indicated as blue letters. The D-region of the heavy chains is shown on white background. Sequence numbering and CDR definitions follow Kabat nomenclature[2].

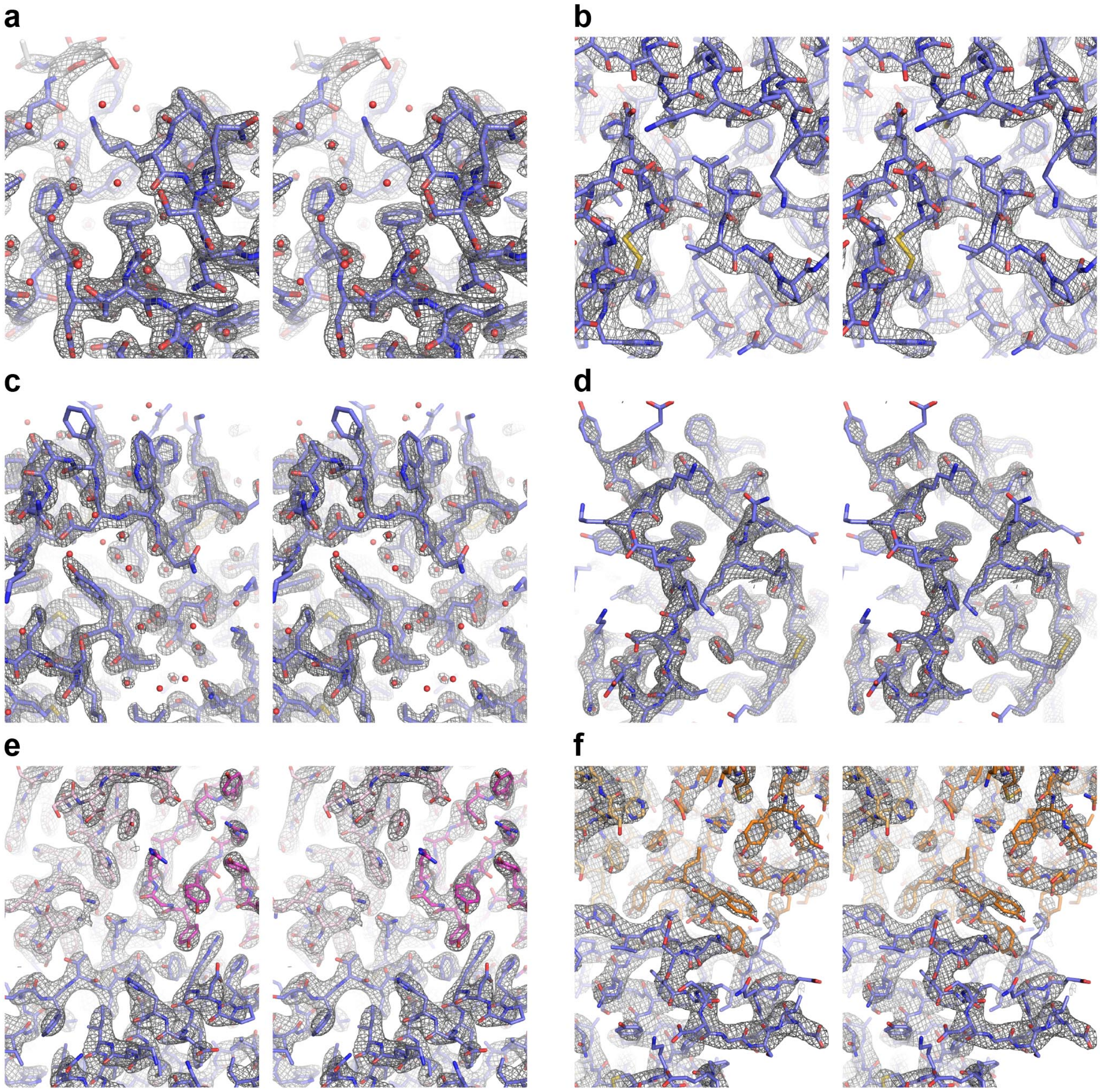

Supplementary Figure 6: Stereo images of portions of the crystallographic 2mFo-DFc electron density maps contoured at 2.0 σ. Related to Methods. **a** SBV Gc head-stalk, **b** BUNV Gc head domain, **c** LACV Gc head domain, **d** OROV Gc head domain, **e** SBV Gc head with scFv 1C11, **f** SBV Gc head with scFv 4B6.

Supplementary Table 1: Crystallographic data collection and refinement statistics for anisotropically processed datasets. Related to Fig. 1, Fig. 5, Fig. 7.

	SBV Gc(465-874)<br><br>PDB: 6H3S	SBV Gc(465-702)<br>with scFv 1C11<br>PDB: 6H3T	SBV Gc(465-702)<br>with scFv 4B6<br>PDB: 6H3U
**Data Collection and Processing**			
Space group	$P\,4_1\,2_1\,2$	$C\,1\,2\,1$	$P\,3_1\,2\,1$
Cell dimensions			
a, *b*, *c* [Å]	95.23, 95.23, 204.74	262.79, 44.40, 93.59	175.84, 175.84, 153.68
α, β, γ [°]	90.00, 90.00, 90.00	90.00, 91.81, 90.00	90.00, 90.00, 120.00
Resolution range[a] [Å]	47.93–2.01 (2.28–2.01)	46.77–2.84 (3.17–2.84)	48.55–3.17 (3.61–3.17)
Ellipsoidal resolution[b] [Å] / direction	2.52 / *a**<br>2.52 / *b**<br>2.02 / *c**	2.80 / 0.97*a** + 0.23*c**<br>3.21 / *b**<br>3.61 / -0.89*a** + 0.46*c**	4.03 / 0.89*a** - 0.45*b**<br>4.03 / *b**<br>3.15 / *c**
R_{merge}[a]	0.10 (1.91)	0.07 (0.81)	0.22 (1.78)
$\langle I/\sigma(I)\rangle$ [a]	20.6 (1.7)	15.3 (2.3)	11.4 (2.0)
$CC_{1/2}$[a]	1.00 (0.66)	1.00 (0.84)	1.00 (0.72)
Completeness, spherical[a] [%]	60.9 (9.8)	64.2 (11.4)	59.1 (9.3)
Completeness, ellipsoidal[a,b] [%]	95.4 (81.0)	90.6 (60.8)	94.1 (70.0)
Redundancy[a]	17.6 (15.6)	6.8 (6.8)	20.9 (18.7)
**Structure Refinement**			
Resolution range[a] [Å]	47.93–2.01 (2.28–2.01)	46.77–2.84 (3.17–2.84)	48.55–3.17 (3.61–3.17)
Number of unique reflections[a]	38,793 (1,940)	16,839 (842)	27,774 (1,391)
R_{work} / R_{free}	0.210 / 0.240	0.213 / 0.262	0.200 / 0.228
Number of atoms			
Protein	6,474	7,282	7,571
Ligands/ions	116	242	242
Water	306	0	0
Average *B*-factor [Å^2]			
Protein	64.99	87.19	111.47
Ligands/ions	79.88	120.99	164.40
Water	55.93	–	–
R.m.s. deviations			
Bond lengths [Å]	0.003	0.004	0.004
Bond angles [°]	0.56	0.82	0.76

[a] Values in parentheses are for the highest-resolution shell.

[b] The datasets were anisotropically truncated using the STARANISO web server. An ellipsoid was fitted to the anisotropic cut-off surface to provide approximate resolution limits along three directions in reciprocal space. The real cut-off surface is only approximately ellipsoidal and the directions of the worst and best resolution limits may not correspond with the reciprocal axes.

Supplementary Table 2: Crystallographic data collection and refinement statistics for isotropically processed datasets. Related to Fig. 2.

	BUNV Gc(478-721) PDB: 6H3V	LACV Gc(477-722) PDB: 6H3W	OROV Gc(482-702) PDB: 6H3X
**Data Collection and Processing**			
Space group	*P* 3 2 1	*H* 3 2	*H* 3 2
Cell dimensions			
a, b, c [Å]	125.44, 125.44, 46.55	73.23, 73.23, 280.71	104.64, 104.64, 133.50
α, β, γ [°]	90.00, 90.00, 120.00	90.00, 90.00, 120.00	90.00, 90.00, 120.00
Resolution range[a] [Å]	46.55–2.90 (3.08–2.90)	47.05–2.10 (2.16–2.10)	44.50–2.09 (2.15–2.09)
R_{merge}[a]	0.09 (1.27)	0.09 (1.02)	0.09 (0.71)
$\langle I/\sigma(I) \rangle$ [a]	16.1 (1.9)	11.4 (1.8)	13.4 (1.7)
$CC_{1/2}$[a]	1.00 (0.70)	1.00 (0.65)	1.00 (0.80)
Completeness[a] [%]	99.8 (99.8)	99.3 (98.0)	99.1 (89.2)
Redundancy[a]	9.6 (10.0)	7.9 (7.6)	11.1 (9.6)
**Structure Refinement**			
Resolution range[a] [Å]	46.55–2.90 (3.08–2.90)	47.05–2.10 (2.16–2.10)	44.50–2.09 (2.15–2.09)
Number of unique reflections[a]	9,534 (1,519)	17,376 (1,378)	16,679 (1,159)
R_{work} / R_{free}	0.167 / 0.216	0.165 / 0.213	0.203 / 0.233
Number of atoms			
Protein	1,958	1,979	1,802
Ligands/ions	39	61	45
Water	0	223	40
Average *B*-factor [Å^2]			
Protein	102.59	30.67	74.62
Ligands/ions	137.17	48.63	118.65
Water	–	36.58	66.25
R.m.s. deviations			
Bond lengths [Å]	0.009	0.007	0.003
Bond angles [°]	1.16	0.95	0.61

[a] Values in parentheses are for the highest-resolution shell.

Supplementary Table 3: Sequence identities and root-mean square deviations among Gc head domain crystal structures. Related to Fig. 2.

	Sequence Identity	RMSD
BUNV:LACV	29 %	2.0 Å (over 232 Cα atoms)
BUNV:OROV	23 %	3.5 Å (over 216 Cα atoms)
BUNV:SBV	17 %	3.5 Å (over 216 Cα atoms)
LACV:OROV	23 %	3.0 Å (over 216 Cα atoms)
LACV:SBV	21 %	3.7 Å (over 224 Cα atoms)
OROV:SBV	21 %	3.1 Å (over 216 Cα atoms)

Supplementary Table 4: Primers for the amplification and restriction-free cloning of codon-optimized synthetic gene fragments. Related to Methods.

		Name	Sequence
pMT backbone for inserts with C-terminal single Strep tag[a,c]	5’	2nd_Strep.Gf	GGTGGATGGTCACACCCTCAATTCGAGAAGT
	3’	BIP-REV	CCCGAGCGAGAGGCCAACAAA
SBV Gc head-Strep[a,c]	5’	SBV_Bip_GcNl.Gf	CCTTTGTTGGCCTCTCGCTCGGGCAGGAAACCAGCATCAACTGC
	3’	SBV_GcNl_Str.Gr	CTCGAATTGAGGGTGTGACCATCCACCAATCAGGCTCAGGGTGGTCAG
scFv 1C11-Strep[a,c]	5’	HC_1C11_1F4.Gf	CCTTTGTTGGCCTCTCGCTCGGGGAGGTTCAGCTGCAGCAGTC
	3’	2nd_Strep.GR	ACTTCTCGAATTGAGGGTGTGACCATCCACC
scFv 4B6-Strep[a,c]	5’	HC_4B6.Gf	CCTTTGTTGGCCTCTCGCTCGGGCAGATGCAGCTTCAGGAGTC
	3’	2nd_Strep.GR	ACTTCTCGAATTGAGGGTGTGACCATCCACC
OROV Gc head-Strep[a,c]	5’	OROV_Bip-482.Gf	CTTTGTTGGCCTCTCGCTCGGGGACGAGGATTGCCTGTCCA
	3’	OROV_702-Str.Gr	GAATTGAGGGTGTGACCATCCACCCAGGTTGATATCCAGCAGCTT
BUNV Gc head-Strep[a,c]	5’	BUNV_Bip-GcN.Gf	CTTTGTTGGCCTCTCGCTCGGGGAGGAGGATTGCTGGAAGAACG
	3’	BUN_GcNl_Str.Gr	CTCGAATTGAGGGTGTGACCATCCACCCAGCTCGGTTGGCTGGTACAG
pMT backbone for inserts with N-terminal single Strep tag[a,c]	5’	pT350_TGA.Gf	TGAGTTTAAACCCGCTGATCAGCCTCGACTG
	3’	pT350_Bip-Str.Gr	CTCCCTTCTCGAATTGTGGATGGCTCCACTCCCCGAGCGAGAGGCCAAC
SBV Gc Strep-head-stalk[a,c]	5’	SBV_Str-465.Gf	AGCCATCCACAATTCGAGAAGGGAGGTCAGGAAACCAGCATCAACTGC
	3’	SBV_874-TGA.Gr	CGAGGCTGATCAGCGGGTTTAAACTCACCACACGATGTCGCTGCAGGAG
LACV Gc Strep-head-stalk[a,c]	5’	LACV_Str-477.Gf	AGCCATCCACAATTCGAGAAGGGAGGTGGCGATTTCACCACCTGCCTGGA
	3’	LACV_911-TGA.Gr	CGAGGCTGATCAGCGGGTTTAAACTCACCAATTGCAGTTGCTGATGTGC
SBV Gc Strep-ectodomain[b,c]	5’	SBV_Str-465.Gf	AGCCATCCACAATTCGAGAAGGGAGGTCAGGAAACCAGCATCAACTGC
	3’	SBV_1326.Gr	GAGGCTGATCAGCGGGTTTAAACTCACTGCTCGATATAGGATGTCTC
SBV Gc head-linker-Strep[b,d]	5’	pMT-GcN_F	TTGTTGGCCTCTCGCTCGGGAGCATCAACTGCAAGAACAT
	3’	pMT-GcN_R	ACGATGTGGGCGGAACCTCCAATCAGGCTCAGGGTGGTCA
	3’	pMT-Linker_R	CCATCACGATGTGGGCTGCAGATCCGCCACCATTTCCTCCGGCGCCTCC-<br>ACCATTACCGTTGCCGCCCCGGCTGTCGGATTGGGAACCTCCAATCAGG
SBV Gc head-stalk-linker-Strep[b,d]	5’	pMT-GcN_F	TTGTTGGCCTCTCGCTCGGGAGCATCAACTGCAAGAACAT
	3’	pMT-Gc874_R	CTGTCGGATTGGGAACCTCCCCACACGATGTCGCTGCAGG
SBV Gc stalk-linker-Strep[b,d]	5’	pMT-Pept2_F	TTGTTGGCCTCTCGCTCGGGCAGACCCTGACCACCCTGAG
	3’	pMT-Gc874_R	CTGTCGGATTGGGAACCTCCCCACACGATGTCGCTGCAGG
SBV Gc core-linker-Strep[b,d]	5’	pMT_P890_F	TTGTTGGCCTCTCGCTCGGGCCCGATATCGAGAACTATAT
	3’	Q1326_Linker_R	CTGTCGGATTGGGAACCTCCCTGCTCGATATAGGATGTCT

[a]To generate constructs used in crystallization.

[b]To generate constructs used in serum depletion analysis.

[c]Cloning was performed by Gibson assembly (New England Biolabs)[3].

[d]Cloning was performed by restriction-free PCR assembly[4].

Supplementary Table 5: Oligonucleotides for RT-qPCR. Related to Methods.

		Name	Sequence
SBV	5’	SBV-S-382F	TCAGATTGTCATGCCCCTTGC
	3’	SBV-S-469R	TTCGGCCCCAGGTGCAAATC
	Probe	SBV-S-408FAM	TTAAGGGATGCACCTGGGCCGATGGT (6-Fam / BHQ-1)
β actin	5’	ACT-1005-F	CAGCACAATGAAGATCAAGATCATC
	3’	ACT-1135-R	CGGACTCATCGTACTCCTGCTT
	Probe	ACT-1081-HEX	TCGCTGTCCACCTTCCAGCAGATGT (Hex / BHQ-1)

Supplementary Table 6: Primers for the amplification of the variable domain and the first constant domain of IgG2a heavy chains (HC) and the variable domain of the light chains (LC). Related to Methods.

		Name	Sequence[a]
		mVH1_1aa	ATTATCGTACGGTTCTTTCCCAGGTYCAGCTGCAGCAGTCTGGASC
		mVH1_1a2	ATTATCGTACGGTTCTCTCCCAGGTTCAGCTGCAGCAGTCTG
		mVH1_1b	ATTATCGTACGGTCCTCTCTGAGGTCCAGCTGCARCAGTCTGGASC
		mVH1_2a	ATTATCGTACGGTTCTCTCCCAGGTYCAGCTGCAGCAGTCTGGR
		mVH1_2b	ATTATCGTACGGTCCTCTCCCAGGTCCAACTGCAGCAGCCTG
		mVH2	ATTATCGTACGGTTTTATCCCAGGTGCAGCTGAAGSAGTCAG
		mVH3	ATTATCGTACGGTCCTGTCTGAKGTRCAGCTTCAGGAGTC
		mVH4	ATTATCGTACGGTTTTATCTGAGGTGAAGCTTCTCGAGTCTGGAGG
		mVH5	ATTATCGTACGGTTCTGAGTGAVGTGMAGCTGGTGGAGTCTG
		mVH6	ATTATCGTACGGTATTGTCTGATGTGAACYTGGAAGTGTCTGGAGGAG
HC variable region	5'	mVH6_2	ATTATCGTACGGTATTATCTGAAGTGAAGCTTGAGGAGTCTGGAGGAG
		mVH7	ATTATCGTACGGTGTTGAGTGAGGTGAAGCTGRTGGARTCTGGAGG
		mVH8	ATTATCGTACGGTGCTATCCCAGGTTACTCTGAAAGAGTCTGGCCCT
		mVH9	ATTATCGTACGGTCCTTTCACAGATCCAGTTGGTGCAGTCTGG
		mVH10	ATTATCGTACGGTTCTTAGTGAGGTGCAGCTTGTTGAGWCTGG
		mVH11	ATTATCGTACGGTGTTGAGTGAAGTGCAGCTGTTGGAGACTGG
		mVH12	ATTATCGTACGGTCCTGTCCCAGATGCAGCTTCAGGAGTC
		mVH13	ATTATCGTACGGTACTTTCCCAGGTGCAGCTTGTAGAGACCGGRG
		mVH14	ATTATCGTACGGTTCTTTCAGAGGTTCAGCTGCAGCAGTCTG
		mVH15	ATTATCGTACGGTGCTATCTCAGGTTCACCTACAACAGTCTGGTTCTGA
		mVH16	ATTATCGTACGGTACTATCGGAGGTGCAGCTGGTGGAATCTGGA
		mVH16_2	ATTATCGTACGGTACTATCGGAGGTGCAGCTGGAGGAGAGTGGA
IgG2a constant region	3'	mIgG2a	ATTACCTAGGTTATTTACCCGGAGTCCGGGAGAAGCTC
		mVk1_1	TATTCCGTACGGATGTTGTGATGACCCAAACTCCACTCTC
		mVk1_2	TAATGCGTACGGATGTTGTGATGACCCAGACTCCACT
		mVk2_1	GATTACGTACGGATATTGTGATGACGCAGGCTGCA
		mVk2_2	GACTGCGTACGGATATTGTGATAACCCAGGATGAACTCTCC
		mVk3_1	ATTATCGTACGGACATTGTGCTRACACAGTCTCCTGCT
		mVk3_2	ACTATCGTACGGACATTGTGCTGACCCAATCTCCAG
		mVk4_1	ATAATCGTACGGAAAATGTGCTCACCCAGTCTCCA
		mVk4_2	CTTCTCGTACGCAAATTGTTCTCACCCAGTCTCCAGCA
		mVk5_1	ATTATCGTACGGACATCBTGCTGACTCAGTCTCCAGC
		mVk5_2	TCAGTCGTACGGATATTGTGCTAACTCAGTCTCCAGCCA
		mVk6_1	TATCTCGTACGGACATTGTGATGACCCAGTCTCAMAAAT
		mVk6_2	ATTCCCGTACGAGTATTGTGATGACCCAGACTCCCAAA
		mVk6_3	CTATCCGTACGAACATTGTAATGACCCAATCTCCCAAAT
LC variable region	5'	mVk4_1	ATAATCGTACGGAGCTCGTGATGACCCAGACCCCA
		mVk7	ATTCTCGTACGGACATTGTGATGACTCAGTCTCCAACTTTC
		mVk8_1	ACCATCGTACGGAAATTGTGTTGACCCAGTCTATACCATCC
		mVk8_2	ATTCTCGTACGGACATTGTGATGWCACAGTCTCCATCCTC
		mVk9	ATTAGCGTACGGACATCCAGATGACCCAGTCTCCATC
		mVk10	ATGCTCGTACGGATATCCAGATGACACAGACTACWTCCTCC
		mVk11	ATTGTCGTACGGATGTCCAGATGATTCAGTCTCCATCCT
		mVk12	ATTCTCGTACGGACATCCAGATGACTCAGTCTCCAGCC
		mVk13	ACCAGCGTACGGACATCCAGATGACACAATCTTCATCCT
		mVk14	ATTAGCGTACGGACATCAAGATGACCCAGTCTCCATCYTC
		mVk15	ATTAGCGTACGGACATCCAGATGAACCAGTCTCCATCC
		mVk16	ATTAGCGTACGGATGTCCAGATAACCCAGTCTCCATCTTAT
		mVk17	ATTATCGTACGGAAACAACTGTGACCCAGTCTCCAGC
		mVk18	ACTAACGTACGACTGGAGAAACAACACAGGCTCCAG

		mVk19	ATTAGCGTACGGACATCCAGATGACACAGTCTCCATCC
		mVk20	AGCAGCGTACGAATATCCAGGTGATCCAGTCACCATTTC
LC variable region	3’	mJk1	ATTATGTCGACWTTGATTTCCAGCTTGGTGCCTCC
		mJk2	ATTATGTCGACTTTTATTTCCAGCTTGGTCCCCG
		mJk3	ATTAGGTCGACTTTTATTTCCAGTCTGGTCCCATCACTG
		mJk4	ATTATGTCGACTTTTATTTCCAAYTTTGTCCCCGWGC
		mJk5	ATTATGTCGACTTTCAGCTCCAGCTTGGTCCCAG

[a] Restriction enzyme cleavage sites are shown in blue. Nucleotides shown in red were inserted to optimize cleavage.

Supplementary References

1. Dolinsky, T. J., Nielsen, J. E., McCammon, J. A. & Baker, N. A. PDB2PQR: an automated pipeline for the setup of Poisson-Boltzmann electrostatics calculations. *Nucleic Acids Res.* **32**, W665-667 (2004).

2. Kabat, E. A., Wu, T. T., Perry, H. M., Foeller, C. & Gottesman, K. S. *Sequences of Proteins of Immunological Interest*. (DIANE Publishing, 1992).

3. Gibson, D. G. *et al.* Enzymatic assembly of DNA molecules up to several hundred kilobases. *Nat. Methods* **6**, 343–345 (2009).

4. Geiser, M., Cébe, R., Drewello, D. & Schmitz, R. Integration of PCR Fragments at Any Specific Site within Cloning Vectors without the Use of Restriction Enzymes and DNA Ligase. *BioTechniques* **31**, 88–92 (2001).
