# Supplementary material for: Orthobunyavirus spike architecture and recognition by neutralizing antibodies
Source: Nat Commun. 2019 Feb 20;10:879. doi: 10.1038/s41467-019-08832-8 (PMC6382863; doi:10.1038/s41467-019-08832-8)
Supplement: Supplementary file 3 — Description of Additional Supplementary Files [file 41467_2019_8832_MOESM3_ESM.docx]

**Description of Supplementary Files**

**File Name:** Supplementary Data 1

**Description:** Amino acid sequence alignment of the N-terminal variable region of Gc from selected Orthobunyaviruses in the Simbu, Bunyamwera and California serogroups. The respective GenBank accession numbers are given in Methods. Related to Supplementary Figure 3d.

**File Name:** Supplementary Data 2

**Description:** Amino acid sequence alignment of the N-terminal variable region of Gc from selected Simbu serogroup Orthobunyaviruses together with all publicly available natural Schmallenberg virus sequences. The respective GenBank accession numbers are provided in Methods. Related to Figure 7.
