# Supplementary material for: Orthobunyavirus spike architecture and recognition by neutralizing antibodies
Source: Nat Commun. 2019 Feb 20;10:879. doi: 10.1038/s41467-019-08832-8 (PMC6382863; doi:10.1038/s41467-019-08832-8)

Orthobunyavirus Gc Variable Region
Selected Sequences

- A divergent residue within group
- A conserved residue within group
- A strictly conserved residue within group
- C cysteine involved in disulfide bond
- N predicted N-glycosylation site
- residue at the 1C11 epitope in SBV
- residue at the 4B6 epitope in SBV
- residue at the trimer interface in BUNV or LACV

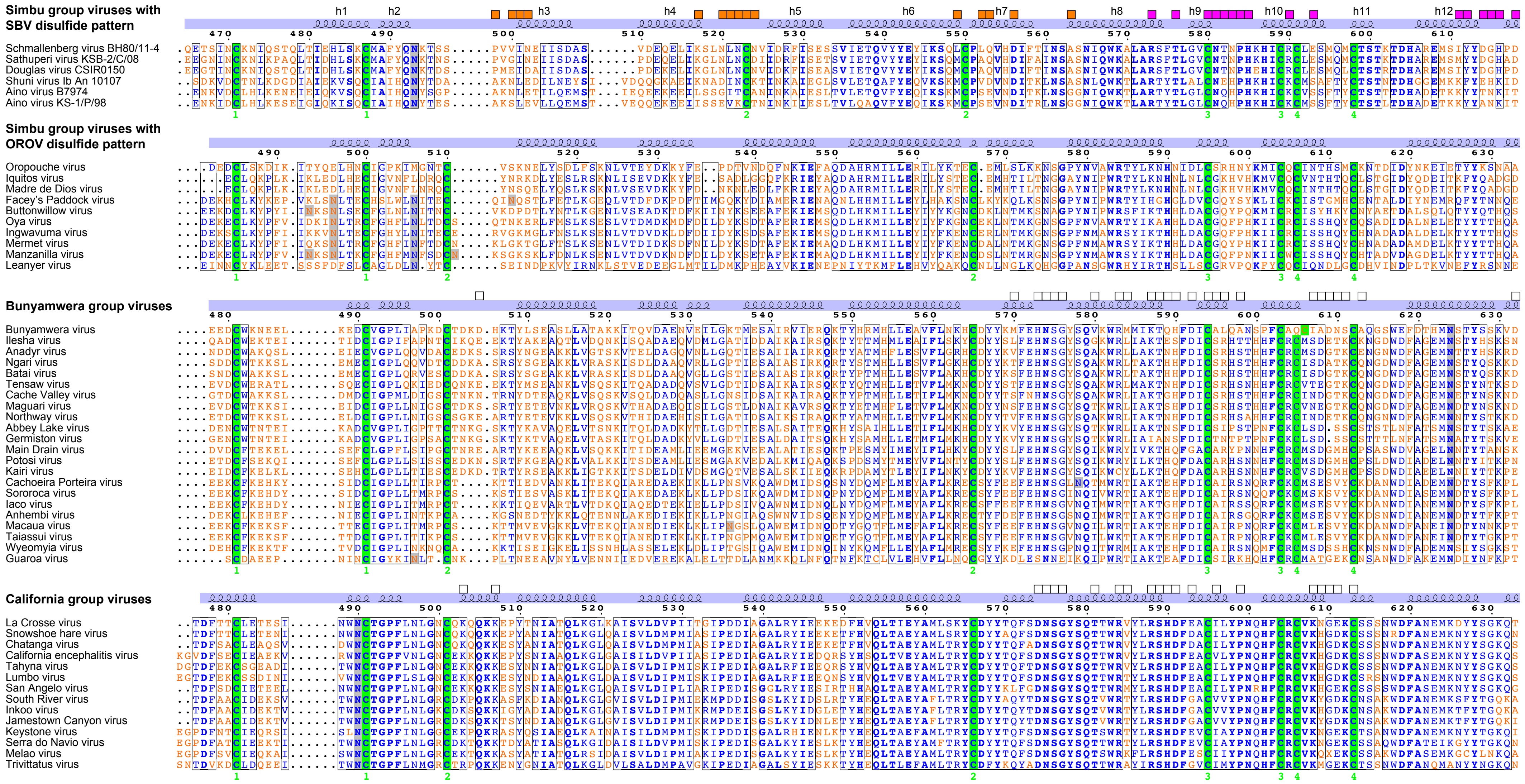

Orthobunyavirus Gc Variable Region Selected Sequences

A divergent residue within group
A conserved residue within group
A strictly conserved residue within group
C cysteine involved in disulfide bond
N predicted N-glycosylation site
residue at the 1C11 epitope in SBV
residue at the 4B6 epitope in SBV
residue at the trimer interface in BUNV or LACV

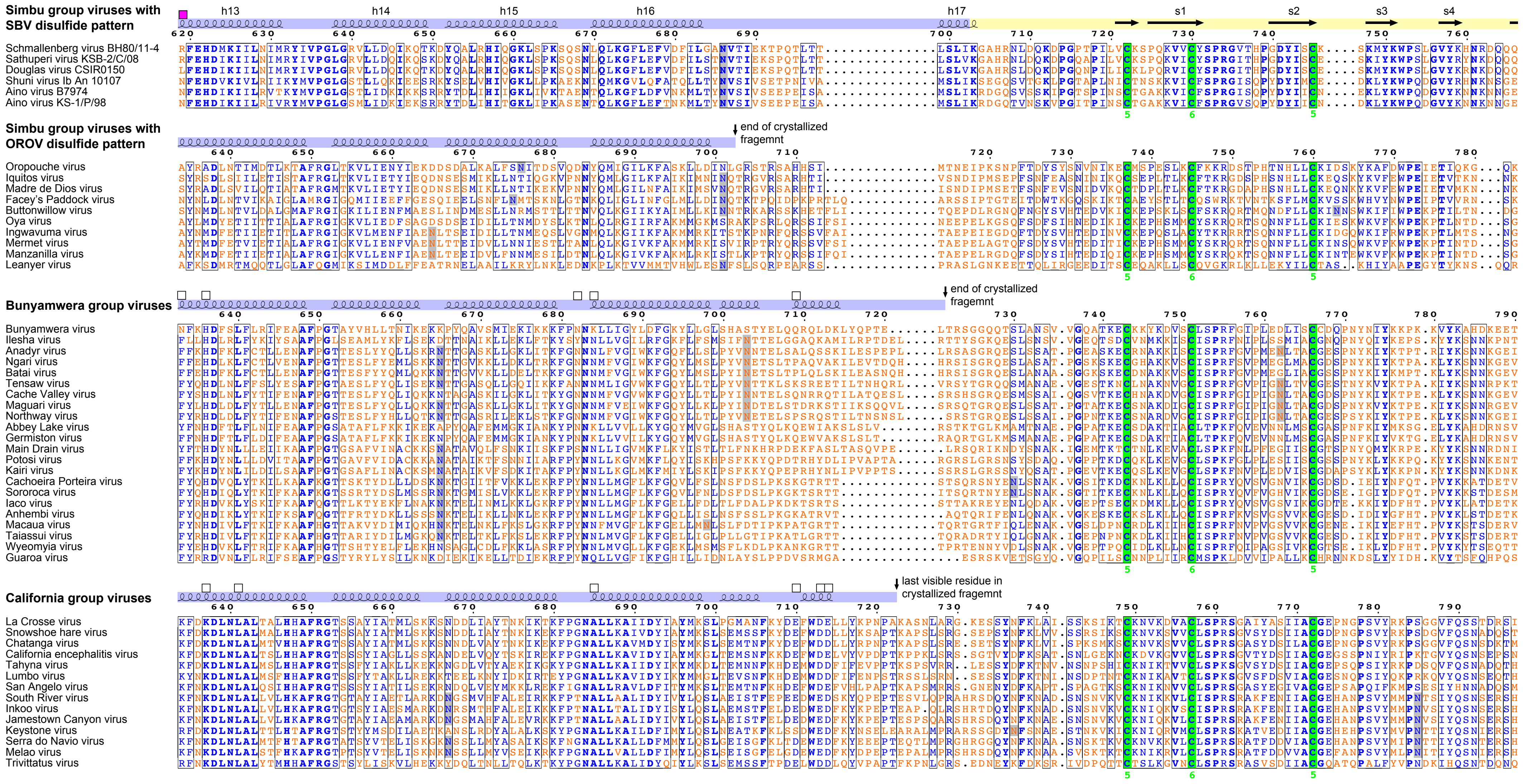

Orthobunyavirus Gc Variable Region
Selected Sequences

- A divergent residue within group
- A conserved residue within group
- A strictly conserved residue within group
- C cysteine involved in disulfide bond
- N predicted N-glycosylation site
- residue at the 1C11 epitope in SBV
- residue at the 4B6 epitope in SBV
- residue at the trimer interface in BUNV or LACV

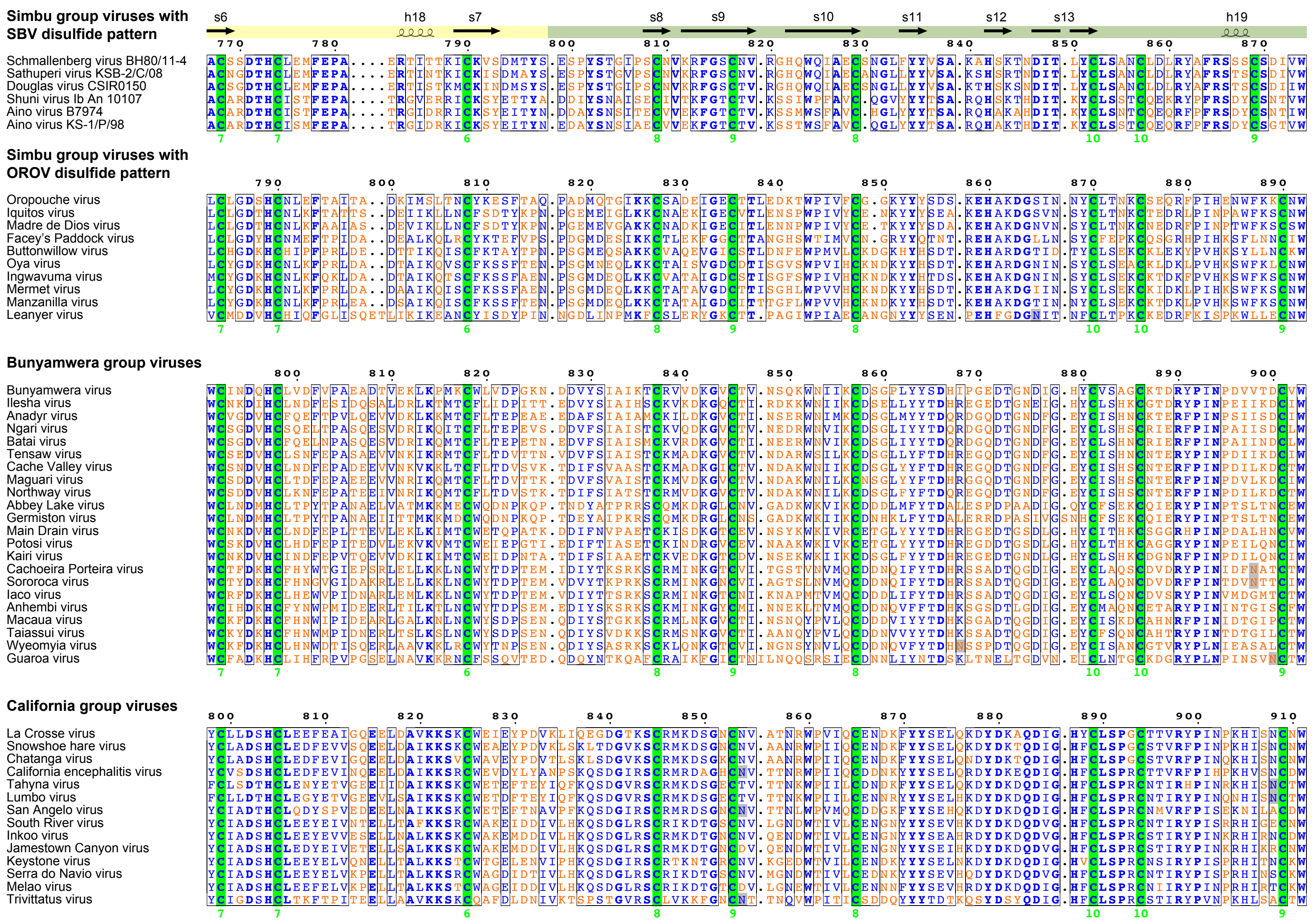

Supplement: Supplementary file 4 — Supplementary Data 1 [file 41467_2019_8832_MOESM4_ESM.pdf]
