# Supplementary material for: Orthobunyavirus spike architecture and recognition by neutralizing antibodies
Source: Nat Commun. 2019 Feb 20;10:879. doi: 10.1038/s41467-019-08832-8 (PMC6382863; doi:10.1038/s41467-019-08832-8)
Supplement: Supplementary file 5 — Supplementary Data 2 [file 41467_2019_8832_MOESM5_ESM.pdf]

Schmallenberg Virus Gc Variable Region Sequence Alignment

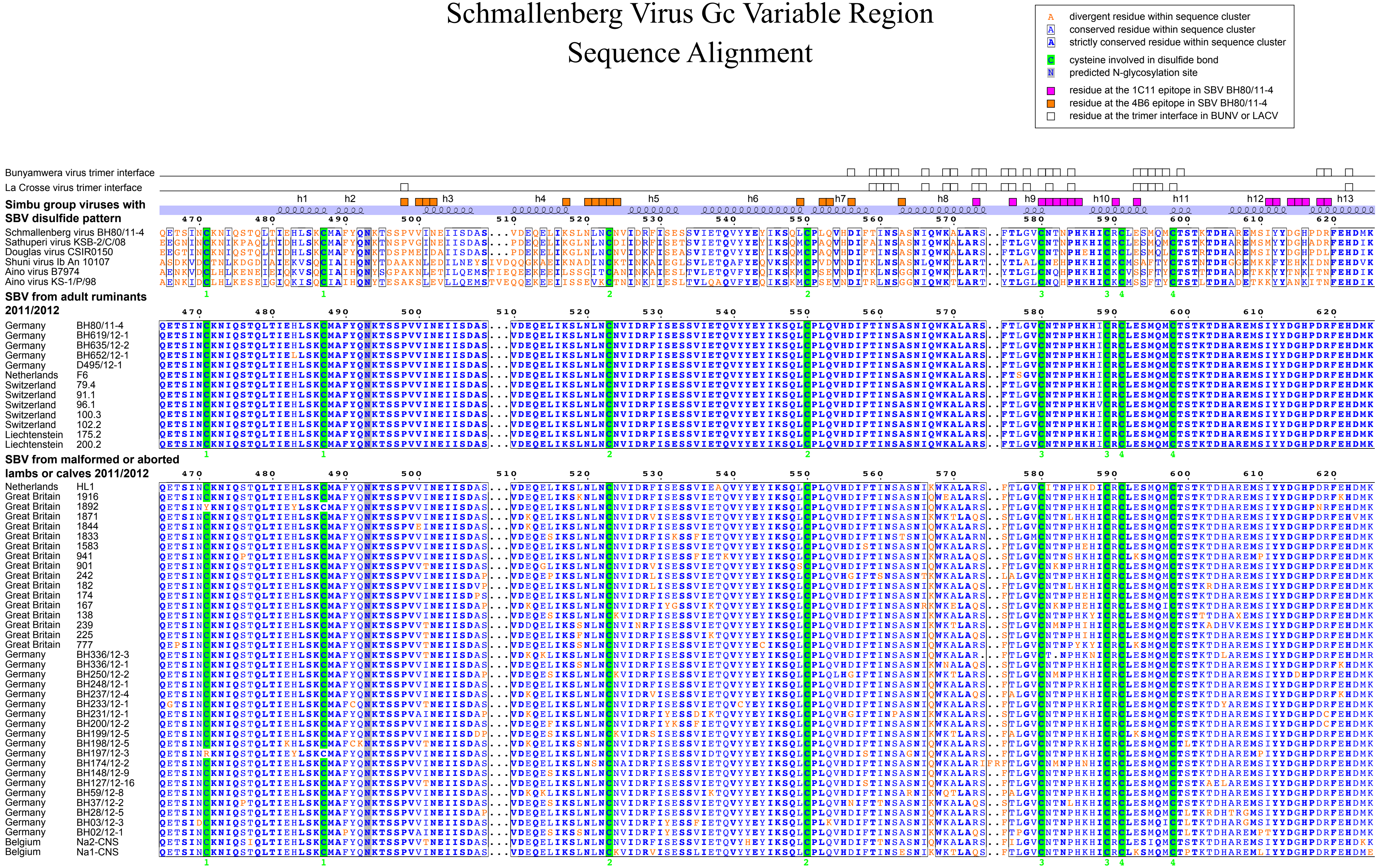

Schmallenberg Virus Gc Variable Region Sequence Alignment

- A divergent residue within sequence cluster
- A conserved residue within sequence cluster
- A strictly conserved residue within sequence cluster
- C cysteine involved in disulfide bond
- N predicted N-glycosylation site
- residue at the 1C11 epitope in SBV BH80/11-4
- residue at the 4B6 epitope in SBV BH80/11-4
- residue at the trimer interface in BUNV or LACV

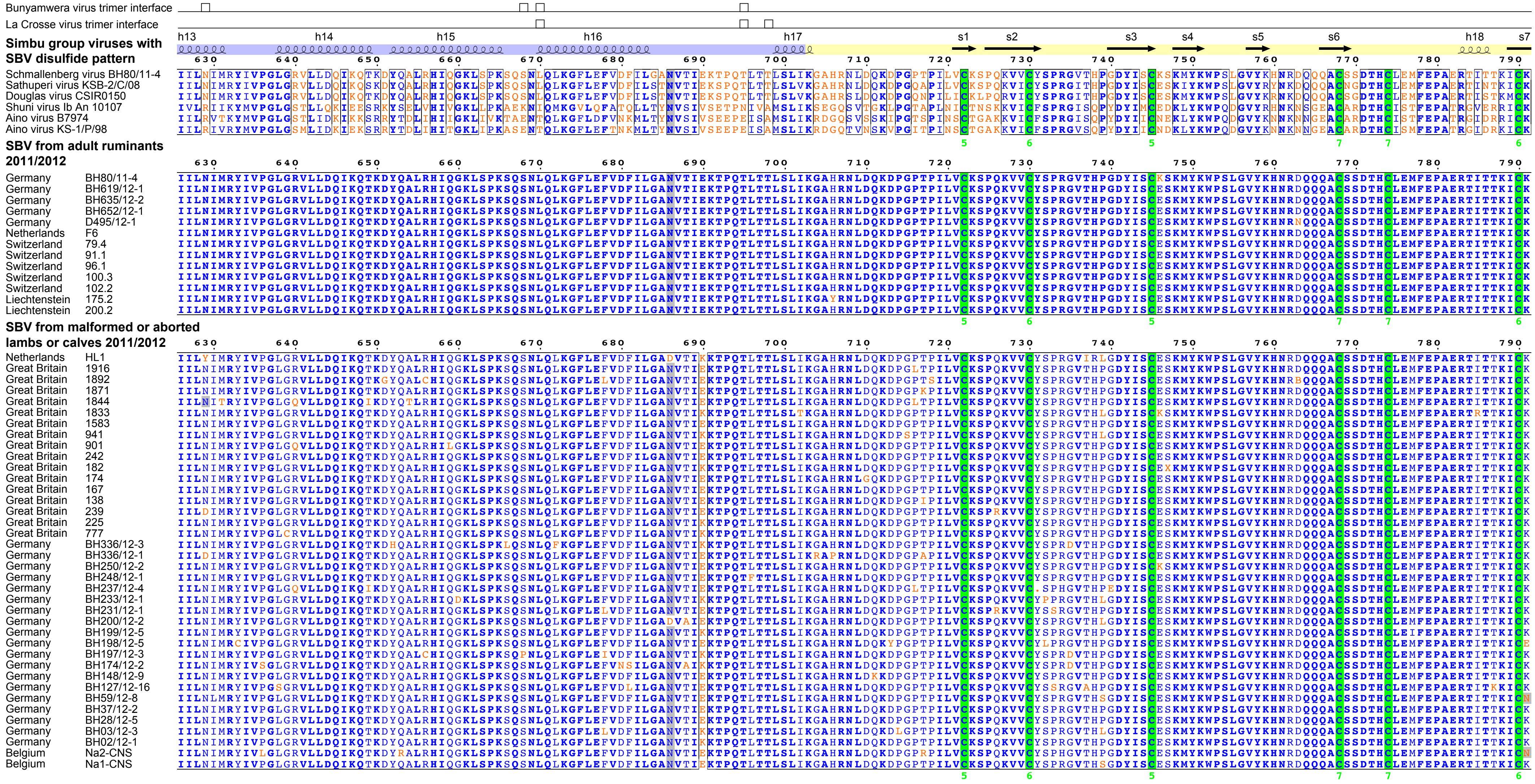

Schmallenberg Virus Gc Variable Region Sequence Alignment

Symbol	Meaning
A (orange)	divergent residue within sequence cluster
A (boxed)	conserved residue within sequence cluster
A (boxed, bold)	strictly conserved residue within sequence cluster
C (green)	cysteine involved in disulfide bond
N (grey)	predicted N-glycosylation site
■ (magenta)	residue at the 1C11 epitope in SBV BH80/11-4
■ (orange)	residue at the 4B6 epitope in SBV BH80/11-4
□	residue at the trimer interface in BUNV or LACV

Bunyamwera virus trimer interface

La Crosse virus trimer interface

Secondary structure: s8, s9, s10, s11, s12, s13, h19

Positions: 800, 810, 820, 830, 840, 850, 860, 870

Simbu group viruses with SBV disulfide pattern

Virus	Sequence
Schmallenberg virus BH80/11-4	VSDMTYSESPYSTGIPSCNVKRFGSCNVRGHQWQIAECSNGLFYYVSAKAHSKTNDITLYCLSANCLDLRYAFRSSSCSDIVW
Sathuperi virus KSB-2/C/08	ISDMAYSESPYSTGVPSCNVKRFGSCNVRGHQWQIAECANGLLYYVSAKSHSRTNDITLYCLSANCLDLRYAFRSTSCSDIVW
Douglas virus CSIR0150	INDMSYSESPYSTGIPSCNVKRFGSCTVRGHQWQIAECSNGLLYYVSAKTHSKSNDITLYCLSANCLDLRYAFRSTSCSDIIW
Shuni virus Ib An 10107	SYETTYADDIYSNAISECIVTKFGTCTVKSSIWPFAVC.QGVYYYTSARQHSKTHDITKYCLSSTCQEKRYPFRSDYCSNTVW
Aino virus B7974	SYEITYNDDAYSNSITECVVEKFGTCTVKSSMWSFAVC.HGLYYYTSARQHAKAHDITKYCLSNTCQEQRFPFRSDYCSNTIW
Aino virus KS-1/P/98	SYEITYNEDAYSNSIAECVVEKFGTCTVKSSTWSFAVC.QGLYYYTSARQHAKTHDITKYCLSSTCQEQRFPFRSDYCSGTVW

Disulfide bonds: 8 9 8 10 10 9

SBV from adult ruminants 2011/2012

Country	Isolate	Sequence
Germany	BH80/11-4	VSDMTYSESPYSTGIPSCNVKRFGSCNVRGHQWQIAECSNGLFYYVSAKAHSKTNDITLYCLSANCLDLRYAFRSSSCSDIVW
Germany	BH619/12-1	VSDMTYSESPYSTGIPSCNVKRFGSCNVRGHQWQIAECSNGLFYYVSAKAHSKTNDITLYCLSANCLDLRYAFRSSSCSDIVW
Germany	BH635/12-2	VSDMTYSESPYSTGIPSCNVKRFGSCNVRGHQWQIAECSNGLFYYVSAKAHSKTNDITLYCLSANCLGLRYAFRSSSCSDIVW
Germany	BH652/12-1	VSDMTYSESPYSTGIPSCNVKRFGSCNVRGHQWQIAECSNGLFYYVSAKAHSKTNDITLYCLSANCLDLRYAFRSSSCSDIVW
Germany	D495/12-1	VSDMTYSESPYSTGIPSCNVKRFGSCNVRGHQWQIAECSNGLFYYVSAKAHSKTNDITLYCLSANCLDLRYAFRSSSCSDIVW
Netherlands	F6	VSDMTYSESPYSTGIPSCNVKRFGSCNVRGHQWQIAECSNGLFYYVSAKAHSKTNDITLYCLSANCLDLRYAFRSSSCSDIVW
Switzerland	79.4	VSDMTYSESPYSTGIPSCNVKRFGSCNVRGHQWQIAECSNGLFYYVSAKAHSKTNDITLYCLSANCLDLRYAFRSSSCSDIVW
Switzerland	91.1	VSDMTYSESPYSTGIPSCNVKRFGSCNVRGHQWQIAECSNGLFYYVSAKAHSKTNDITLYCLSANCLDLRYAFRSSSCSDIVW
Switzerland	96.1	VSDMTYSESPYSTGIPSCNVKRFGSCNVRGHQWQIAECSNGLFYYVSAKAHSKTNDITLYCLSANCLDLRYAFRSSSCSDIVW
Switzerland	100.3	VSDMTYSESPYSTGIPSCNVKRFGSCNVRGHQWQIAECSNGLFYYVSAKAHSKTNDITLYCLSANCLDLRYAFRSSSCSDIVW
Switzerland	102.2	VSDMTYSESPYSTGIPSCNVKRFGSCNVRGHQWQIAECSNGLFYYVSAKAHSKTNDITLYCLSANCLDLRYAFRSSSCSDIVW
Liechtenstein	175.2	VSDMTYSESPYSTGIPSCNVKRFGSCNVRGHQWQIAECSNGLFYYVSAKAHSKTNDITLYCLSANCLDLRYAFRSSSCSDIVW
Liechtenstein	200.2	VSDMTYSESPYSTGIPSCNVKRFGSCNVRGHQWQIAECSNGLFYYVSAKAHSKTNDITLYCLSANCLDLRYAFRSSSCSDIVW

Disulfide bonds: 8 9 8 10 10 9

SBV from malformed or aborted lambs or calves 2011/2012

Country	Isolate	Sequence
Netherlands	HL1	VSDMTYSESPYSTGIPSCNVKRFGSCNVRGHQWQIAECSNGLFYYVSAKAHSKTNDITLYCLSANCLDLRYAFRSSSCSDIVW
Great Britain	1916	VSDMTYSESPYSTGIPSCNVKRFGSCNVRGHQWQIAECSNGLFYYVSAKAHSETNNITLYCLSANCLDLRYAFRSSSCSDIVW
Great Britain	1892	VSDMTYSESPYSTGIRSCNVKRFGSCNVRGHQWQIAECSNGLFYYVSAKAHSETNDITLYCLSANCLDLRYAFRSSSCSDIVW
Great Britain	1871	VSDMTYSESPYSTGIPSCNVKRFGSCNVRGHQWQIAECSNGLFYYVSAKAHSKTNNITLYCLSANCLDLHYAFRSSSCSDIVW
Great Britain	1844	VSDMTYSESPYSTGIPSCNVKRFGSCNVRGHQWQIAECSNGLFYYVSAKAHSKTNNITLYCLSANCLDLRYAFRSSSCSDIVW
Great Britain	1833	VSDMTYSESPYSTGIPSCNVKRFGSCNVRGHQWQIAECSNGLFYYVSAKAHSKTNDITLYCLSANCLDLRYAFRSSSCSDIVW
Great Britain	1583	VSDMTYSESPYSTGIPSCNVKRFGSCNVRGHQWQIAECSNGLFYYVSAKAHSKTNDITLYCLSANCLDLRYAFRSSSCSDIVW
Great Britain	941	VSDMTYSESPYSTGIPSCNVKRFGSCNVRGHQWQIAECSNGLFYYVSAKAHSKTNDITLYCLSANCLDLRYAFRSSSCSDIVW
Great Britain	901	VSDMTYSESPYSTGIPSCNVKRFGSCNVRGHQWQIAECSNGLFYYVSAKAHSKTNDITLYCLSANCLDLRYAFRSSSCSDIVW
Great Britain	242	VSDMTYSESPYSTGIPSCNVKRFGSCNVRGHQWQIAECSNGLFYYVSAKAHSETNDITLYCLSANCLDLRYAFRSSSCSDIVW
Great Britain	182	VSDMTYSESPYSTGIPSCNVKRFGSCNVRGHQWQIAECSNGLFYYVSAKAHSKTNDITLYCLSANCLDLRYAFRSSSCSDIVW
Great Britain	174	VSDMTYSESPYSTGIPSCNVKRFGSCNVRGHQWQIAECSNGLFYYVSAKAHSKTNDITLYCLSANCLDLRYAFRSSSCSDIVW
Great Britain	167	VSDMTYSESPYSTGIPSCNVKRFGSCNVRDHQWQIAECSNGLFYYVSAKAHSKTNDITLYCLSANCLDLRYAFRSSSCSDIVW
Great Britain	138	VIDMTYSESPYSTGIPSCNVKRFGSCNVRGHQWQIAECSNGLFYNVSAKAHSKTNDITLYCLSANCLDLRYAFRSSSCSDIVW
Great Britain	239	VSDMTYSKSPYSTGILSCNVKRFGSCNVRGHQWQIAECSNGLFYYVSAKAHSKTNDITLYCLSANCLDLRYAFRSSSCSDIVW
Great Britain	225	VSDMTYSESPYSTGIPSCNVKRFGSCNVRGHQWQIAECSNGLFYYVSAKAHSKTNDITLYCLSANCLDLRYAFRSSSCSDIVW
Great Britain	777	VSDMTYSESPYSTGIPSCNVKRFGSCNVRDHQWQIAECSNGLFYYVSAKAHSKTNDITLYCLSANCLDLRYAFRSSSCSDIVW
Germany	BH336/12-3	VSDMTYSESPYSTGILSCNVKRFGSCNVRGHQWQIAKCSNGLFYYVSAKAHSKTNDITLYCLSANCLDLRYAFRSSSCSDIVW
Germany	BH336/12-1	VSDMTYSESPYSTGIPSCNVKRFGSCNVRGHQWQIAECSNGLFYYVSAKAHSKTNDVTLYCLSANCLDLHYAFRSSSCSDIVW
Germany	BH250/12-2	VIDMTYSESPYSTGIPSCNVKRFGSCNVRGHQWQIAECSNGLFYYVSARAHSKTNDITLYCLSANCLDLRYAFRSSSCSDIVW
Germany	BH248/12-1	VSDMTYSESPYSTGIPSCNVKRFGSCNARGHQWQIAECSNGLFYYVSAKAHSKTNDITLYCLSANCLDLCYAFRSSSCSDILW
Germany	BH237/12-4	VSDMTYSESPYSTGIPSCNVKRFGSCNVRGHQWQIAECSNGLFYYVSAKAHSKTNNITLYCLSANCLDLRYAFRSSSCSDIVW
Germany	BH233/12-1	VSDMAYSESPYSTGIPSCNVKRFGSCNVRGHQWQIAECSNGLFYYVSAKAHSKTNDITLYCLSANCLDLRYAFRSSSCSDIVW
Germany	BH231/12-1	VSDMTYSESPYSTGIPSCNVKRFGSCNVRGHQWQIAECSNGLFYYVSAKAHSKTNDITLYCLSANCLDLRYAFRSSSCSGIVW
Germany	BH200/12-2	VSDMTYSESPYSTGIPSCNVKRFGSCNVRGHQWQIAECSNGLFYYVSAKAHSKTNDITLYCLSANCLDLRYAFRSSSCSDIVW
Germany	BH199/12-5	VSDMTYSESPYSIGIPSCNVKRFGSCNVRGHQWQIAECSNGLFYYVSAKAHSETNDITLYCLSANCLDLRYAFRSSSCSDIVW
Germany	BH198/12-5	VSDMTYSESPYSTGIRSCNVKRFGSCNVRGHQWQIAECSNGLFYYVSAKAHSKTNDITLYCLSANCLDLRYAFRSSSCSDIVW
Germany	BH197/12-3	VSDMTYSESPYSTGIPSCNVKRFGSCNVGGHQWQIAECSNGLFYYVSAKAHSKTNDITLYCLSANCQDLRYAFRSSSCSDIVW
Germany	BH174/12-2	VSDMTYSESPYSTGLPSCNVKRFGSCNVRGHQWQIAECSNGLFYYVSAKAHSKTNNITLYCLSANCLDLRYAFRSSSCSDIVW
Germany	BH148/12-9	VSDMTYSESPYSTGISSCNVERFGSCNVRGHQWQIAECSNGLFYYVSAKAHSKTNDITLYCLSANCLDLCYAFRSSSCSDIVW
Germany	BH127/12-16	VSDMTYSESPYSTGIPSCNVKRFGSCNVRGHQWQIAECSNGLFYYVSAKAHSKTNDITLYCLSANCLDLRYAFRSSSCSDIVW
Germany	BH59/12-8	VSDMTYSESPYSTGIPSCNVKRFGSCNVRGHQWQIAECSNGLFYYVSAKAHSKTNDITLYCLSANCLDLRYAFRSSSCSDIVW
Germany	BH37/12-2	VSDMTYSESPYSTGIPSCNVKRFGSCNVRGHQWQIAECSNGLFYYVSAKAHSKTNDITLYCLSANCLDLRYAFRSSSCSDIVW
Germany	BH28/12-5	ASDMTYSESPYSTGIPSCNVKRFGSCNVRGHQWQIAECSNGLFYYVSAKAHSKTNDITLYCLSANCLDLRYAFRSSSCSDIVW
Germany	BH03/12-3	VSDMTYSKSPYSTGIPSCNVKRFGLCNVRGHQWQIAECSNGLFYYVSAKAHSKTNDITLYCLSANCLDLRYAFRSSSCSDIVW
Germany	BH02/12-1	VSDMTYSESPYSTGIPSCNVKRFGSCNVRGHQWQIAECSNGLFYYVSAKAHSETNDITLYCLSANCLDLRYAFRSSSCSDIVW
Belgium	Na2-CNS	VSDMTYSESPYSTGMPSCNVKRFGSCNVRGHQWQIAECSNGLFYYVSAKAHSKTNDITLYCLSANCLDLRYAFRSSSCSDIVW
Belgium	Na1-CNS	VSDMTYSESPYSTGIPSCNVKRFGSCNVRGHQWQIAECSNGLFYYVSAKAHSKTNDITLYCLSANCLDLRYAFRSSSCSDIVW

Disulfide bonds: 8 9 8 10 10 9
