# Supplementary material for: Orthobunyavirus spike architecture and recognition by neutralizing antibodies
Source: Nat Commun. 2019 Feb 20;10:879. doi: 10.1038/s41467-019-08832-8 (PMC6382863; doi:10.1038/s41467-019-08832-8)
Supplement: Supplementary file 6 — Reporting Summary [file 41467_2019_8832_MOESM6_ESM.pdf]

Corresponding author(s): Martin Beer, Félix A. Rey

Reporting Summary

Nature Research wishes to improve the reproducibility of the work that we publish. This form provides structure for consistency and transparency in reporting. For further information on Nature Research policies, see Authors & Referees and the Editorial Policy Checklist.

Statistical parameters

When statistical analyses are reported, confirm that the following items are present in the relevant location (e.g. figure legend, table legend, main text, or Methods section).

n/a	Confirmed	
☐	☒	The exact sample size (n) for each experimental group/condition, given as a discrete number and unit of measurement
☐	☒	An indication of whether measurements were taken from distinct samples or whether the same sample was measured repeatedly
☐	☒	The statistical test(s) used AND whether they are one- or two-sided<br>*Only common tests should be described solely by name; describe more complex techniques in the Methods section.*
☒	☐	A description of all covariates tested
☐	☒	A description of any assumptions or corrections, such as tests of normality and adjustment for multiple comparisons
☐	☒	A full description of the statistics including central tendency (e.g. means) or other basic estimates (e.g. regression coefficient) AND variation (e.g. standard deviation) or associated estimates of uncertainty (e.g. confidence intervals)
☐	☒	For null hypothesis testing, the test statistic (e.g. F, t, r) with confidence intervals, effect sizes, degrees of freedom and P value noted<br>*Give P values as exact values whenever suitable.*
☒	☐	For Bayesian analysis, information on the choice of priors and Markov chain Monte Carlo settings
☒	☐	For hierarchical and complex designs, identification of the appropriate level for tests and full reporting of outcomes
☒	☐	Estimates of effect sizes (e.g. Cohen's d, Pearson's r), indicating how they were calculated
☐	☒	Clearly defined error bars<br>*State explicitly what error bars represent (e.g. SD, SE, CI)*

Our web collection on statistics for biologists may be useful.

Software and code

Policy information about availability of computer code

Data collection	X-ray data collection:<br>MXCuBE2 (http://mxcube.github.io/mxcube/)<br><br>ELISA acquisition:<br>Tecan i-control (https://lifesciences.tecan.com/)<br><br>RT-qPCR acquisition:<br>CFX Maestro Software, Security Edition (http://www.bio-rad.com/)<br><br>Multi-angle static light scattering:<br>Wyatt Technology ASTRA 6 (https://www.wyatt.com/)<br><br>Biolayer Interferometry:<br>ForteBio Octet Data Acquisition 10.0 (https://www.fortebio.com/)
Data analysis	Structure determination:<br>XDS Nov 1, 2016 (http://xds.mpimf-heidelberg.mpg.de/)<br>Aimless 0.5.32 (http://www.ccp4.ac.uk/)<br>Global Phasing Limited STARANISO 1.0.5 web server (http://staraniso.globalphasing.org/)

SHELXE 2016/1 (http://shelx.uni-goettingen.de/)
Phenix 1.10.1-2155 (https://www.phenix-online.org/)
Coot 0.8.8-pre EL (https://www2.mrc-lmb.cam.ac.uk/personal/pemsley/coot/)

Structure analysis:
Dali web server (http://ekhidna.biocenter.helsinki.fi/dali_server/)
PDBePISA 1.52 web server (http://www.ebi.ac.uk/pdbe/pisa/)
Phyre2 web server (http://www.sbg.bio.ic.ac.uk/phyre2/)

Structure representation:
PyMOL 1.7.4.4 (https://pymol.org/)
UCSF Chimera 1.11.2 (http://www.rbvi.ucsf.edu/chimera)
PDB2PQR 2.1.1 web server (http://nbcr-222.ucsd.edu/pdb2pqr_2.1.1/)

Sequence alignments and conservation analysis:
Clustal Omega web server (https://www.ebi.ac.uk/Tools/msa/clustalo/)
ESPript 3.0 web server (http://espript.ibcp.fr/)
IMGT/V-QUEST web server (http://www.imgt.org/IMGT_vquest/share/textes/)

Multi-angle static light scattering:
Wyatt Technology ASTRA 6 (https://www.wyatt.com/)

Biolayer interferometry:
ForteBio Octet Data Analysis HT 10.0 (https://www.fortebio.com/)

Focus Reduction Neutralization Assay:
Nikon NIS-Elements Imaging Basic Research (https://www.nikoninstruments.com/)
QuantumSoft pro Fit 7 (https://www.quansoft.com/)

Statistical analysis:
Systat Software SigmaPlot 11 (https://systatsoftware.com/)
Microsoft Excel 2011 (https://www.microsoft.com/)

For manuscripts utilizing custom algorithms or software that are central to the research but not yet described in published literature, software must be made available to editors/reviewers upon request. We strongly encourage code deposition in a community repository (e.g. GitHub). See the Nature Research guidelines for submitting code & software for further information.

Data

Policy information about availability of data

All manuscripts must include a data availability statement. This statement should provide the following information, where applicable:

- Accession codes, unique identifiers, or web links for publicly available datasets
- A list of figures that have associated raw data
- A description of any restrictions on data availability

Atomic coordinates, structure factor amplitudes, and the respective protein sequences have been deposited in the Protein Data Bank. Accession numbers are PDB: 6H3S for SBV Gc(465-874), PDB: 6H3T for SBV Gc(465-702) / scFv 1C11, PDB: 6H3U for SBV Gc(465-702) / scFv 4B6, PDB: 6H3V for BUNV Gc(478-721), PDB: 6H3W for LACV Gc(477-722) and PDB: 6H3X for OROV Gc(482-702). The source data underlying Figs 3, 4, 5a, 5b, and 6 are provided as a Source Data file. All other relevant data are available from the authors upon request.

Field-specific reporting

Please select the best fit for your research. If you are not sure, read the appropriate sections before making your selection.

☒ Life sciences ☐ Behavioural & social sciences ☐ Ecological, evolutionary & environmental sciences

For a reference copy of the document with all sections, see nature.com/authors/policies/ReportingSummary-flat.pdf

Life sciences study design

All studies must disclose on these points even when the disclosure is negative.

Sample size	The number of animals required for each group was determined using a Chi-square test without a Bonferroni correction.<br>The selected sample size is sufficient to allow comparison of each vaccinated/treated group with the respective mock control group.
Data exclusions	No data were excluded from the analyzes.
Replication	All experiments were performed in duplicates. If the experimental findings could not be replicated using two independent measurements, the experiment was repeated several times.
Randomization	Animal trials: animals were randomly assigned to the different groups. Animals of different gender and age were distributed equally over all

Randomization	groups.<br>For all remaining experiments within the study no comparative analyzes were performed between different groups. Thus, randomization was not relevant.
Blinding	Animal trials: Grouping of animals and assignment to the different experimental groups was not performed by the same person.<br>For the remaining experiments, blinding was not possible since they were designed and performed by the same person.

Reporting for specific materials, systems and methods

Materials & experimental systems

n/a	Involved in the study
☐	☒ Unique biological materials
☐	☒ Antibodies
☐	☒ Eukaryotic cell lines
☒	☐ Palaeontology
☐	☒ Animals and other organisms
☒	☐ Human research participants

Methods

n/a	Involved in the study
☒	☐ ChIP-seq
☒	☐ Flow cytometry
☒	☐ MRI-based neuroimaging

Unique biological materials

Policy information about availability of materials

Obtaining unique materials	All unique materials are available from the authors upon request or can be obtained from standard commercial sources as indicated in the Methods section.

Antibodies

Antibodies used	Immunofluorescence analysis was performed using the original hybridoma supernatant of monoclonal antibody 1C11, specific for SBV Gc, at a dilution of 1:100 in PBS + 0.05% Tween. An anti-mouse Alexa-488-labeled secondary antibody was diluted 1:1000 and used for detection (Molecular Probes, Invitrogen, catalogue number: A-11017).<br>Recombinant mouse monoclonal antibodies IgG2a 1C11 and IgG2a 4B6 targeting the Gc head domain of Schmallenberg virus were prepared as described in the Methods section. No other primary antibodies were used in this study.
Validation	Our recombinant antibodies were tested for reactivity with wildtype virus using immunofluorescence microscopy on infected cells and for reactivity in ELISA with the recombinant Gc head domain of Schmallenberg virus.

Eukaryotic cell lines

Policy information about cell lines

Cell line source(s)	For recombinant protein production:<br>Drosophila S2 cells - Thermo Fisher Scientific - Cat# R690-07<br>FreeStyle 293-F cells - Thermo Fisher Scientific - Cat# R790-07<br><br>For the microneutralization assay:<br>BHK-21 cells - Collection of Cell Lines in Veterinary Medicine, Friedrich-Loeffler-Institut, Greifswald - Insel Riems, Germany<br><br>For the focus reduction neutralization assay:<br>Vero 76 cells - Collection of Cell Lines in Veterinary Medicine, Friedrich-Loeffler-Institut, Greifswald - Insel Riems, Germany
Authentication	Authentication of BHK-21 and Vero 76 cells was performed by the department responsible for the Collection of Cell Lines in Veterinary Medicine at the Friedrich-Loeffler-Institut, Insel Riems.<br>No authentication was performed for cell lines which were only used for recombinant protein production.
Mycoplasma contamination	The BHK-21 and Vero 76 cell lines were tested negative for Mycoplasma contamination.<br>Cells for recombinant protein production were not tested for Mycoplasma contamination.
Commonly misidentified lines<br>(See ICLAC register)	None

Animals and other organisms

Policy information about studies involving animals; ARRIVE guidelines recommended for reporting animal research

Laboratory animals	C57BL/6 IFNAR-/- mice (B6.129S2-Ifnar1tm1Agt/Mmjax)<br>Source: specific pathogen-free breeding unit, Friedrich-Loeffler-Institut, Greifswald - Insel Riems, Germany<br>The animals had mixed sex and were between 5 weeks and 10 months old.
Wild animals	This study did not involve wild animals.
Field-collected samples	This study did not involve samples collected from the field.
